# Uptake in the Central Nervous System of Geraniol Oil Encapsulated in Chitosan Oleate Following Nasal and Oral Administration

**DOI:** 10.3390/pharmaceutics11030106

**Published:** 2019-03-03

**Authors:** Maria Cristina Bonferoni, Luca Ferraro, Barbara Pavan, Sarah Beggiato, Elena Cavalieri, Paolo Giunchedi, Alessandro Dalpiaz

**Affiliations:** 1 Department of Drug Sciences, University of Pavia, Viale Taramelli 12, 27100 Pavia, Italy; elena.cavalieri01@universitadipavia.it; 2 Department of Life Sciences and Biotechnology, University of Ferrara, via Borsari 46, 44121 Ferrara, Italy; frl@unife.it (L.F.); sarah.beggiato@unife.it (S.B.); 3 Department of Biomedical and Specialist Surgical Sciences, University of Ferrara, via Borsari 46, 44121 Ferrara, Italy; pvnbbr@unife.it; 4 Department of Chemistry and Pharmacy, University of Sassari, Via Muroni 23/a, 07100 Sassari, Italy; pgiunc@uniss.it; 5 Department of Chemical and Pharmaceutical Sciences, University of Ferrara, via Fossato di Mortara 19, 44121 Ferrara, Italy

**Keywords:** geraniol, emulsified formulations, amphiphilic chitosan, oleic acid, oral and nasal administration, brain targeting

## Abstract

The pharmacological activities of geraniol include anticancer and neuroprotective properties. However, its insolubility in water easily induces separation from aqueous formulations, causing administration difficulties. Here we propose new emulsified formulations of geraniol by using the amphiphilic polymer chitosan-oleate (CS-OA) as surfactant to combine mucoadhesive and absorption enhancer properties with stabilization effects on the oil dispersion. The formulation based on CS-OA 2% (*w*/*w*) (G-CS-OA-2.0%) showed viscosity values compatible with oral and nasal administration to rats, and mean diameter of the dispersed phase of 819 ± 104 nm. G-CS-OA-2.0% oral administration sensibly increases the geraniol bioavailability with respect to coarse emulsions obtained without CS-OA (AUC values in the bloodstream were 42,713 ± 1553 µg∙mL^−1^∙min and 2158 ± 82 µg∙mL^−1^∙min following administration of 50 mg/kg or 1 mg/kg, respectively), and enhances the aptitude of geraniol to reach the central nervous system from the bloodstream (AUC values in the cerebrospinal fluid were 7293 ± 408 µg∙mL^−1^∙min and 399 ± 25 µg∙mL^−1^∙min after oral administration of 50 mg/kg or 1 mg/kg, respectively). Moreover, relevant geraniol amounts were detected in the cerebrospinal fluid following the G-CS-OA-2% nasal administration (AUC values in the cerebrospinal fluid were 10,778 ± 477 µg∙mL^−1^∙min and 5571 ± 290 µg∙mL^−1^∙min after nasal administration of 4 mg/kg or 1 mg/kg, respectively).

## 1. Introduction

Geraniol (3,7-dimethylocta-trans-2,6-dien-1-ol) is an acyclic monoterpene whose presence is abundant in essential oils extracted from aromatic plants, such as rose, lavender, and lemongrass [1,2]. A wide spectrum of pharmacological activities, including antimicrobial [3], anti-inflammatory [2], antioxidant [4], neuroprotective [5], and anticancer [6] properties, are known to be exerted by geraniol. For instance, it exerts antimicrobial activity by preventing colitis-associated dysbiosis and, after its oral administration, appears able to blunt systemic inflammation of colitic mice [7]. Interestingly, several Gram–negative bacteria (*Enterobacter aerogenes, Escherichia coli, Pseudomonas aeruginosa*)-overexpressing efflux pumps, with consequent multi-drug resistance against antibiotics, restore their drug susceptibility in the presence of geraniol [8,9].

In terms of the activity of geraniol in the central nervous system (CNS), it is known its ability to increase dopaminergic neurons survival after free radical injury. Interestingly, this effect was observed in mice following oral administration. Geraniol is therefore considered among the natural compounds potentially able to act as prophylactic agent against neurodegenerative disorders [10].

Finally, geraniol shows anticancer activity against a wide range of tumors such as pulmonary, pancreatic, renal, and hepatic cancers. These effects were demonstrated either in vitro or in vivo following geraniol oral administration [6].

It is known that geraniol is able to easily cross Caco-2 cell monolayers, suggesting good bioavailability following its oral administration [11]. Very recently we have evidenced, by using human normal colonic epithelial NCM460 cell monolayers, that geraniol permeability from intestinal lumen to blood in vitro simulated domains is about six-fold higher than in the opposite direction. Moreover, we have also observed a very high oral bioavailability of geraniol in rats [12]. 

On the other hand, the administration of geraniol appears often related to formulation problems. Indeed, this compound is characterized by very poor water solubility (100 mg/L at 25 °C) and a relatively high n-octanol/water partition coefficient (2.65) [13]. Being liquid at room temperature, the geraniol insolubility in water easily induces its separation from aqueous formulations, with consequent difficulties of administration by oral or intravenous routes. Several formulations have been proposed in order to try to overcome this problem, such as soy lecithin suspensions [7], emulsions in the presence of glycerol for oral administration [12], or a geraniol dispersion in a DMSO and physiologic solution mixture, in the presence of sodium taurocholate, for intravenous administration [12]. However, the stability of these dispersed systems did not appear ever appropriate in order to guarantee a uniform dispersion of geraniol in the formulations, with its propensity to separate from aqueous phase being very high. In this case, geraniol can reach local high concentrations able to irritate the mucosa [7]. 

Chitosan is widely used in pharmaceutical delivery systems aimed to mucosal and transmucosal applications. This is due to the fact that chitosan, among other advantages, possesses good mucoadhesion properties due to its positive charge, and acts as a permeation enhancer [14,15]. Oleic acid is also well known for its permeation enhancement properties [16]. Recently, a chitosan salt of chitosan and oleic acid, chitosan oleate (CS-OA), has been proposed as amphiphilic polymer to obtain polymeric micelles [17,18] and to strongly stabilize nanoemulsions obtained by a spontaneous emulsification procedure [19,20,21]. Accordingly, the chitosan oleate emulsions obtained, ranging in dimensions between about 250 and 700 nm, showed strongly positive zeta potential, in line with the presence of chitosan at their surface, and mucoadhesive properties. It was however observed that the dimensions of the nanoemulsion droplets increased with the increase of the oil concentration [20]; even if concentration strategies such as ultrafiltration can be envisaged to increase concentrations [21], they can be not suitable for labile and volatile oils. 

The aim of the present study was to evaluate whether new emulsified formulations of geraniol encapsulated in CS-OA salt allow to optimize the geraniol oral administration and favor geraniol delivery to nasal olfactory area, thus inducing its absorption through the nose-to-brain route. To these purposes, CS-OA salt, chosen to exploit the mucoadhesion properties of chitosan and the absorption enhancement effect of both chitosan and oleic acid, was prepared in organic medium, dried as previously proposed [22], and used as polymeric surfactant to encapsulate geraniol without the addition of further low molecular weight surfactants. This formulative approach should combine the advantages of chitosan as a mucoadhesive and absorption enhancer with the stabilization effect that the amphiphilic derivative has on the oil dispersion.

## 2. Materials and Methods

### 2.1. Chemicals

Geraniol (98%), carbazole, low molecular weight (LMW) chitosan (CS; deacetylation degree 80%), HPLC-grade methanol, acetonitrile, ethyl acetate, and water were acquired from Sigma-Aldrich (Milan, Italy). Oleic acid (OA) was from Fluka (Milan, Italy). Male Sprague−Dawley rats were provided by Charles-River (Milan, Italy).

### 2.2. Encapsulation of Geraniol in CS-OA

Here, 1.5 g of low molecular weight chitosan base (Sigma Aldrich, Milan, Italy) were wetted with 0.3 mL of acetic acid and 7.5 mL of ethanol. A few mL of ethanol were added, containing dissolved an amount of oleic acid calculated as 1:1 molar ratio with respect to chitosan theoretical free amino groups, taking into account the declared deacetylation degree (about 80%). Thus, 1.4 g of oleic acid were used per each g of chitosan. The solvents were evaporated in rotavapor at a mild temperature of 30–35 °C. The powder was collected, washed twice with a few mL of ethanol to remove the excess of oleic acid, and dried at room temperature. It was used without further purification. A FTIR analysis of the obtained CS-OA was performed by means of a FTIR-4100 apparatus (Jasco Corporation, Cremella, Lecco, Italy). 

The geraniol oil (in amount corresponding to 5% *w*/*w*) final concentration was introduced in a vial together with an equal amount of water and with the CS-OA in amount corresponding to final concentration either 2% or 3.5% (*w*/*w*). The resulting formulations are hereafter indicated as G-CS-OA-2.0% and G-CS-OA-3.5%, respectively. The remaining amount of bidistilled filtered water (0.22 μm, Sartorius Stedim Biotech GmbH, Göttingen, Germany) was slowly added under stirring by means of an Ultra-Turrax T25 (Janke & Kunkel, IKA^®^ Labortechnik, Germany) equipped with an 8-mm probe (S 25 N-G8). Homogenization was performed at 20,500 rpm for 15 min. Acetic acid was added until final pH of 4.5. Samples of 5 mL final volume were prepared.

### 2.3. Dimensional Characterization

The dimensions of the dispersed phase were analyzed by means of Photon Correlation Spectroscopy (PCS) (N5 Submicron Particle Size Analyzer Beckman Coulter, Milan, Italy). PCS analysis was performed at 30° and at 90° angle of detection of the scattered light. For the analysis the samples were diluted in 0.22-µm filtered bi-distilled water. Zeta potential of the dispersion was measured by means of a Zetasizer Z5 90 (Malvern, Milan, Italy).

### 2.4. Viscosity Measurements

Viscosity was measured by a rotational rheometer (Rheostress RS600, Haake, Karlsruke, Germany), equipped with a cone-plate C35/1 (Ø 35 mm, angle 1°) measuring system. After 180 s of thermostatation, the measurements were carried out in the 10–300 s^−1^ shear rate range at 37 °C. 

### 2.5. HPLC Analysis

Geraniol was quantified by HPLC. The chromatographic apparatus consisted of a modular system (model LC-10 AD VD pump and model SPD-10A VP variable wavelength UV−Vis detector; Shimadzu, Kyoto, Japan) and an injection valve with a 20-µL sample loop (model 7725; Rheodyne, IDEX, Torrance, CA, USA). Separations were performed at room temperature on a 5-µm Hypersil BDS C-18 column (150 mm × 4.6 mm i.d.; Alltech Italia Srl, Milan, Italy) equipped with a guard column packed with the same Hypersil material. Data acquisition and processing were performed on a personal computer using CLASS-VP Software, version 7.2.1 (Shimadzu Italia, Milan, Italy). The detector was set at 210 nm; the mobile phase consisted of an isocratic mixture of water and acetonitrile at a ratio of 60:40 (*v*/*v*). The retention times obtained were 10.5 min for geraniol and 16.0 min for carbazole, used as internal standard for the quantification of geraniol in blood samples (see below).

The chromatographic precision, represented by relative standard deviations (RSDs), was evaluated by repeated analysis (*n* = 6) of the same sample dissolved in a mixture of acetate buffer 100 mM (pH = 4) and acetonitrile 5:95 (*v*/*v*) containing geraniol at a concentration of 500 µM (77.1 μg/mL). The RSD value was 0.88%. Geraniol was quantified by the peak area correlated with the predetermined standard curve over the range 10–1000 µM (1.54–154 μg/mL). The calibration curve was linear (*n* = 9, *r* = 0.995, *p* < 0.001). 

For cerebrospinal fluid (CSF) simulation, standard aliquots of balanced solution (PBS Dulbecco’s without calcium and magnesium) in the presence of 0.45 mg/mL BSA were employed [23,24]. For the geraniol assay in CSF, the chromatographic precision was evaluated by repeated analysis (*n* = 6) of the same sample solution containing 500 µM (77.1 μg/mL) geraniol (RSD = 0.96%) in simulated CSF, and calibration curves of peak areas versus concentration were generated in the range of 50–2000 µM (7.71–309 μg/mL) (*n* = 8, *r* = 0.994, *p* < 0.001).

Recovery experiments of 500 µg/mL (77.1 µg/mL) geraniol from rat whole blood were performed by comparison of the peak areas extracted from blood test samples (see below) at 4 °C (*n* = 6) with those obtained by injection of an equivalent concentration of analyte dissolved in its mobile phase. The average recovery ± S.D. was 65.8 ± 3.1%. The concentrations of this compound were therefore referred to as peak area ratio with respect to the internal standard carbazole. The precision of the method, evaluated by replicate analyses (*n* = 6) of rat blood samples containing 77.1 µg/mL geraniol extracted in the presence of the internal standard (carbazole), was demonstrated by the RSD value of 1.14%. Calibration standards were prepared by spiking blood samples with known amounts of geraniol corresponding to blood concentrations in the range 13–7800 µM, (2.0–1200 μg/mL) at 4 °C and then extracted in the presence of the internal standard (carbazole) as below described (see Section 2.7). The samples were analyzed by HPLC and the calibration curve of peak area ratios versus concentrations was linear (*n* = 10, *r* = 0.996, *p* < 0.001). 

### 2.6. Geraniol Content in Geraniol/CS-OA Emulsion Samples

Buffer acetate (100 mM; pH =4) was added (45 µL) to samples of G-CS-OA-2% or G-CS-OA-3.5% (100 µL) and after vortex acetonitrile (855 µL) was added. Then, the samples were further diluted 1:10 with the mixture of acetonitrile–buffer acetate 95:5 (*v*/*v*). After filtration across regenerated cellulose filters (0.45 µm) the samples were injected into the HPLC system for the geraniol detection. Preliminary experiments indicated that the excipients of samples G-CS-OA-2.0% or G-CS-OA-3.5% did not interfere with the geraniol retention time. The geraniol content in the samples was evaluated for three withdrawals and it was indicated as mg/mL and percentage *v*/*v*.

About the stability studies of the geraniol content in the dispersions, the samples G-CS-OA-2.0% or G-CS-OA-3.5% were stored at 4° C for three months, and then their geraniol content was analyzed as above described.

### 2.7. Administration to Rats of Geraniol/CS-OA Emulsion Samples

Adult male Sprague−Dawley rats (200–250 g body weight) fasted for 24 h received an oral gavage dose of 1 mg/kg (about 250 µg/kg; *n* = 4) or 50 mg/kg geraniol (about 12.5 mg/rat; *n* = 4), contained in 20 µL or 1 mL, respectively, of G-CS-OA-2.0% sample. At the end of the oral administration blood samples (100 µL) were serially collected at 15, 30, 45, 60, and 90 min, and CSF samples (50 µL) were serially withdrawn at fixed time points ranging from 30 and 90 min from each rat, using the cysternal puncture method described by van den Berg et al. [25], which requires a single needle stick and allows the collection of serial (40–50 µL) CSF samples that are virtually blood-free [26]. A total volume of approximately 150 µL of CSF was collected during the experimental session. The CSF samples (10 µL) were immediately injected into the HPLC system for geraniol detection. The blood samples were extracted, immediately hemolyzed in Eppendorf tubes prefilled with 500 mL of water (HPLC grade, about 4 °C), and then 50 µL of 3M sodium hydroxide and 50 µL of internal standard solution (200 µM carbazole dissolved in a H_2_O-MeOH mixture 50:50 *v*/*v*) were added. The samples were extracted twice with 1 mL of water-saturated ethyl acetate. After centrifugation (10 min at 13,000 × *g*), the organic layer was reduced to dryness by N_2_ flow, then 150 µL of a H_2_O:CH_3_CN mixture (50:50 *v*/*v*) were added and, after centrifugation, 10 µL were injected into the HPLC system for geraniol and carbazole detection. 

Nasal administration of geraniol was performed on anesthetized rats laid on their backs. The nasally administered doses were 1 (*n* = 4) or 4 mg/kg (*n* = 4) obtained by introducing an aliquot (10 or 40 μL, respectively) of sample G-CS-OA-2.0% in each nostril of the rats using a semiautomatic pipet which was attached to a short polyethylene tubing. The tubing was inserted approximately 0.6–0.7 cm into each nostril. After the administration, blood (100 μL) and CSF samples (50 μL) were serially collected at fixed time points from each rat, and analyzed as described above.

The blood or CSF geraniol concentrations at the programmed time points were detected with at least four rats, following each type of oral administration. In particular, blood withdrawals for all programmed time points were repeated four times with a corresponding number of rats; CSF withdrawals for all programmed time points were repeated at least four times with a number of rats allowing no more than three CSF withdrawals from each of them.

The area under the concentration curves of geraniol in the rat bloodstream or CSF (AUC, µg mL^−1^ min) following oral or nasal administrations were calculated using the trapezoidal method. All the calculations were performed using the computer program Graph Pad Prism.

### 2.8. Statistical Analysis

Statistical evaluations of particle sizes were performed by means of Statgraphics 5.0, Statistical Graphics Corporation, MD, USA. Differences were determined according to a Student *t*-test, and were considered significant at *p* < 0.05.

Statistical comparisons between AUC values were performed by one way ANOVA followed by Bonferroni’s multiple comparison post-test. *p* < 0.05 was considered statistically significant. The calculations were performed by using the computer program Graph Pad Prism.

## 3. Results

### 3.1. G-CS-OA Delivery System Characterization

**Figure 1:**
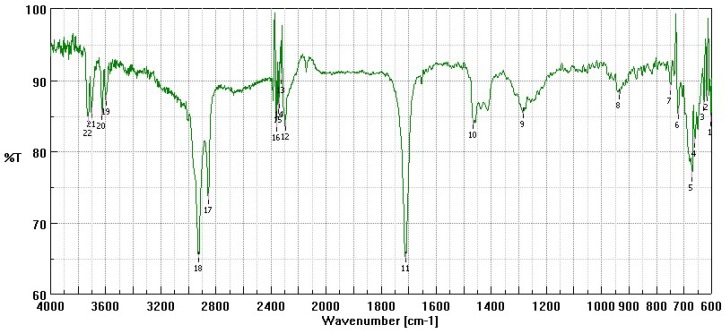
FT-IR spectrum of chitosan-oleate (CS-OA).

Figure 1 shows a FT-IR spectrum performed as preliminary characterization of CS-OA used to encapsulate the geraniol oil. It is possible to see the peaks between 2800 and 3200 cm^−1^ attributable to the oleic acid C=C groups. No evidence of amidic group signals occurrence can be seen, suggesting the electrostatic nature of the interaction between polysaccharide amino groups and oleic acid. 

**Figure 2:**
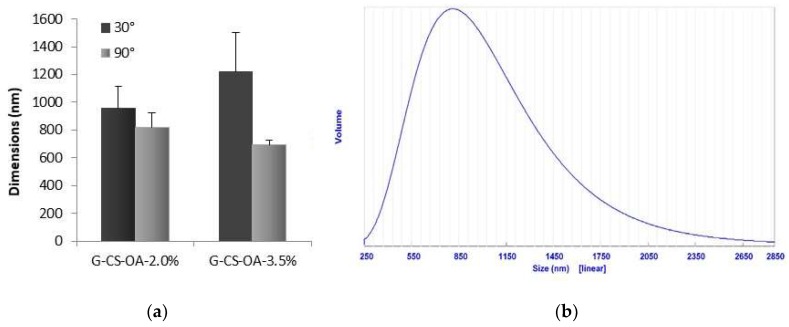
Dimensional characterization of the dispersed phase of geraniol in o/w emulsions. (**a**) mean values obtained from volume distributions (mean ± S.D., *n* = 3), (**b**) example of volume distribution recorded at 90° of detection angle (G-CS-OA-2.0% sample).

Figure 2 shows the mean diameters obtained by Photon Correlation Spectroscopy for the two systems prepared at 2.0% and at 3.5% (*w*/*w*) of CS-OA, at 30° and at 90° degrees of detection. One example of particle size distribution is given too. As the measurements performed at the lowest detection angle are more sensitive to the presence of large particles, the highest values obtained in these conditions correspond to the presence of few particles of larger dimensions, in accordance with the shown distribution (Figure 2b). The values obtained, although slightly below the micrometer limit, are relatively high with respect to those typical of nanoemulsions, that usually have dimensions below 300 nm [27]. This result is however in line with what previously observed in dispersions stabilized by CS-OA with lemongrass oil concentration higher than 0.5% (*w*/*w*) [19], as it was noticed that the increase in oil and CS-OA concentration resulted in the increase of the dimensions of the dispersed phase. In accordance with the results previously obtained on lemongrass nanoemulsions, in the present case the zeta potential value was of 70.4 ± 0.5 mV (mean ± S.D., *n* = 3), confirming the presence of a chitosan shell around the essential oil droplets. 

**Figure 3:**
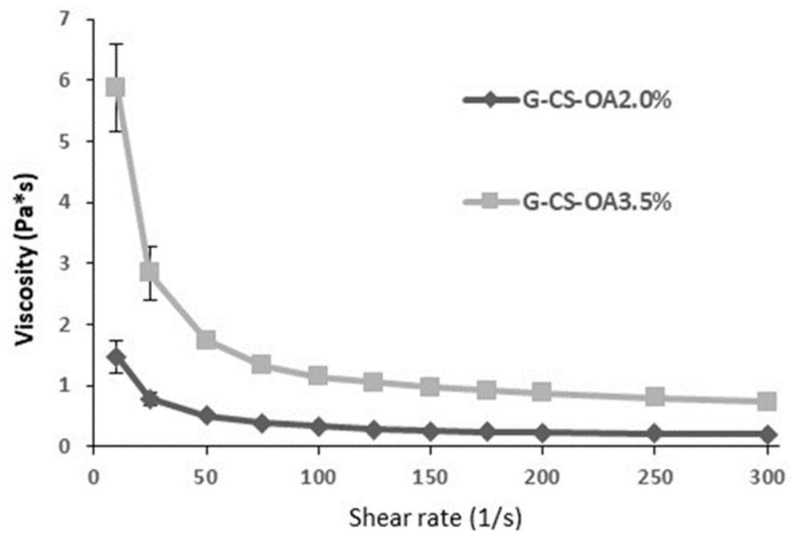
Viscosity curves of the emulsions based on CS-OA-2% and CS-OA-3.5% (*w*/*w*) (mean ± S.D., *n* = 3).

The use of a surfactant of polymeric nature is moreover responsible for relatively high viscosity of the dispersion, as can be seen in Figure 3, where the viscosity curves for both the samples are illustrated. In particular, the viscosity values of the sample based on CS-OA-3.5% (*w*/*w*) resulted unsuitable for oral or nasal administration for its too high viscosity, so the work was continued with the G-CS-OA-2.0% sample. No formal stability evaluation was performed in this work, although for the selected sample the particle size was assessed after storage at 4 °C for one month to confirm that no substantial modification occurred between the preparation and the in vivo administration. The difference in observed dimensions, from 819 ± 104 nm to 957 ± 115 nm (mean ± S.D., *n* = 3), resulted not statistically significant (*t*-Student test, *p* = 0.0596). A partial creaming effect was observed, but resuspension occurred easily after agitation. To verify the sensibility of the sample to an increase of temperature as it can occur at the moment of the administration, the particle size was assessed also after 24 h of storage at 37 °C, and resulted equal to 893 ± 138 nm (mean ± S.D., *n* = 3), in line with the values obtained just after preparation and after 1 month at 4 °C.

### 3.2. Geraniol Content in Geraniol/CS-OA Emulsion Samples

Geraniol content in G-CS-OA-2.0% formulation was 13.1 ± 1.2 mg/mL, corresponding to 1.54 ± 0.14% (*v*/*v*). Similar values were found for CS-OA 3.5% formulation, being the geraniol content 12.6 ± 1.0 mg/mL, corresponding to 1.43 ± 0.12% (*v*/*v*). Geraniol contents in G-CS-OA 2.0% and G-CS-OA 3.5% formulations stored three months at 4 °C did not significantly change during time (data not shown).

### 3.3. In Vivo Administration

Geraniol was administered at the oral doses of 1 mg/kg or 50 mg/kg, and at the intranasal doses of 1 mg/kg or 4 mg/kg.

**Figure 4:**
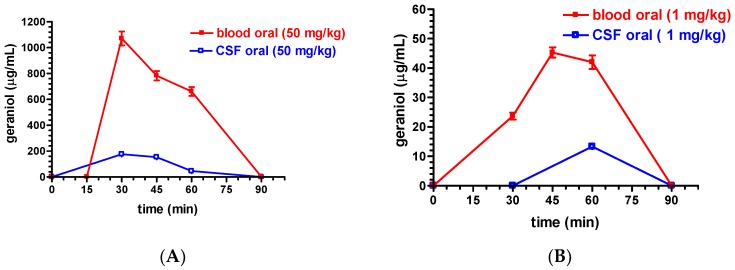

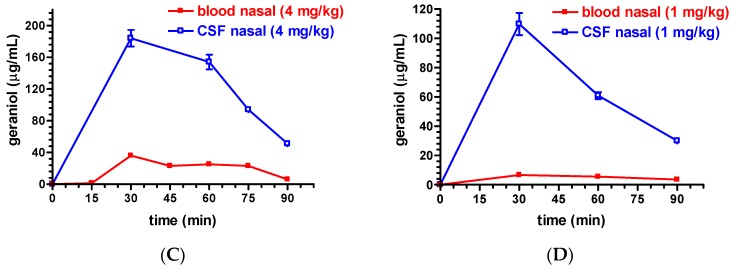
(**A**) Rat blood and cerebrospinal fluid (CSF) geraniol concentrations (µg/mL) within 90 min after oral administration of 50 mg/kg geraniol formulated as G-CS-OA-2.0% emulsion. (**B**) Rat blood and CSF geraniol concentrations (µg/mL) within 90 min after oral administration of 1 mg/kg geraniol formulated as G-CS-OA-2.0% emulsion. (**C**) Rat blood and CSF geraniol concentrations (µg/mL) within 90 min after nasal administration of 4 mg/kg geraniol formulated as G-CS-OA-2.0% emulsion. (**D**) Rat blood and CSF geraniol concentrations (µg/mL) within 90 min after nasal administration of 1 mg/kg geraniol formulated as G-CS-OA-2.0% emulsion. Data are expressed as the mean ± S.D. of at least four independent experiments.

Figure 4A reports the rat blood and CSF geraniol concentrations detected at different time points after the oral administration of the G-CS-OA-2.0% emulsion at the geraniol dosages of 50 mg/kg. A maximum concentration peak (*C*_max_) of 1071 ± 93 µg/mL was obtained in the blood 30 min after the oral administration, then the geraniol concentrations decreased to 783 ± 64 µg/mL and 661 ± 59 µg/mL at 45 and 60 min, respectively, and, finally, negligible amounts were detected 90 min after the administration. On the other hand, a maximum concentration peak (*C*_max_) of 176 ± 20 µg/mL was obtained in the CSF 30 min after the oral administration, and then the geraniol concentrations decreased to 153 ± 18 µg/mL and 46 ± 8 µg/mL at 45 and 60 min, respectively; finally, negligible CSF geraniol amounts were detected 90 min after its administration.

The AUC value obtained for rat blood within 90 min after the oral administration of 50 mg/kg of geraniol formulated as G-CS-OA-2.0% emulsion was 42,713 ± 1553 µg·mL^−1^ min (Table 1), showing a value significantly higher (*p* < 0.001) than the AUC value obtained in the CSF (7293 ± 408 µg·mL^−1^ min, Table 1).

Figure 4B reports the rat blood and CSF geraniol concentrations detected at different time points after the oral administration of 1 mg/kg geraniol formulated as the G-CS-OA-2.0% emulsion. A maximum concentration peak (*C*_max_) of 45 ± 3 µg/mL was obtained in the blood 45 min after the administration, following the value of 23 ± 2 µg/mL obtained at 30 min. The geraniol concentration was maintained to about 40 µg/mL 60 min after the administration, and then no geraniol amounts was detected at 90 min. On the other hand, a concentration value of 13.3 ± 0.12 µg/mL was obtained in the CSF 60 min after the administration, while no geraniol amounts were detected at 30 or 90 min time points. 

The AUC value obtained for rat blood within 90 min after the oral administration of 1 mg/kg of geraniol formulated as G-CS-OA-2.0% emulsion was 2158 ± 82 µg⋅mL^−1^ min (Table 1), showing a value significantly higher (*p* < 0.05) than the AUC value obtained in the CSF (399 ± 25 µg⋅mL^−1^ min, Table 1).

Figure 4C reports the rat blood and CSF geraniol concentrations detected at different time points after the nasal administration of 4 mg/kg geraniol formulated as G-CS-OA-2.0% emulsion. The rat blood geraniol concentrations were 36 ± 5 µg/mL and 23 ± 4 µg/mL 15 and 75 min after administration; moreover, 6 µg/mL were detected at the 90 min time point. On the other hand, a maximum concentration peak (*C*_max_) of 184 ± 18 µg/mL was obtained in the CSF 30 min after the administration, then the concentration values decreased during time relatively slowly (154 ± 16, 94 ± 9 and 51 ± 4 µg/mL at 45, 60 and 90 min, respectively). 

The AUC value obtained for rat blood within 90 min after the nasal administration of 4 mg/kg of geraniol formulated as G-CS-OA-2.0% emulsion was 1668 ± 101 µg⋅mL^−1^ min (Table 1), showing a value significantly lower (*p* < 0.001) than the AUC value obtained in the CSF (10,778 ± 477 µg⋅mL^−1^ min, Table 1).

Figure 4D reports the rat blood and CSF geraniol concentrations detected at different time points after the nasal administration of 1 mg/kg geraniol formulated as G-CS-OA-2.0% emulsion. The rat blood geraniol concentrations ranged between 6.6 ± 1 µg/mL and 3.6 ± 0.3 µg/mL 30 and 90 min after the administration. On the other hand, a maximum concentration peak (*C*_max_) of 110 ± 38 µg/mL was obtained in the CSF 30 min after the administration, thereafter the concentration values decreased during time (61 ± 3 and 30 ± 2 µg/mL 60 and 90 min after the administration, respectively). 

The AUC value obtained for rat blood within 90 min after the nasal administration of 1 mg/kg of geraniol formulated as G-CS-OA-2.0% emulsion was 419 ± 23 µg⋅mL^−1^ min (Table 1), showing a value significantly lower (*p* < 0.001) than the AUC value obtained in the CSF (5571 ± 290 µg⋅mL^−1^ min, Table 1).

The ratios between CSF/blood AUC values were 0.17 or 6.4 following the oral (50 mg/kg) or the nasal (4 mg/kg) geraniol administration, respectively, whereas they were 0.18 or 13.3 following the oral or nasal administration of 1 mg/kg of geraniol, respectively (Table 1).

## 4. Discussion

Despite the high propensity of geraniol to be absorbed in the bloodstream by crossing intestinal cell barriers [12], formulative problems can be related to its administration, both by intravenous and oral routes. Indeed, the geraniol poor water solubility and its liquid state at room temperatures induce a marked tendency of geraniol to separate from the aqueous medium with consequent poor stability of emulsified or suspended systems [7].

Here we propose a new emulsified formulation obtained in the presence of an amphiphilic polymer, CS-OA as surfactant where geraniol was dispersed as droplets below the micron limit. In particular, the CS-OA 2.0% sample showed a mean size of particles of about 800 nm that do not statistically increase after sample storage at 4 °C for a month, as reported in paragraph 3.1, evidencing the good physical stability of this emulsified system. With respect to previous experience on CS-OA-based nanoemulsion preparation [19,20], obtained by means of spontaneous emulsification process with in situ occurrence of CS-OA, the employment of previously prepared CS-OA and the use of a high energy dispersion method resulted less effective in terms of the reduction of droplet size. This seemed in the present case not to affect the physical stability of the dispersion at least during the period in which it was assessed. This result could be attributed to the efficient stabilization of o/w interfaces by means of polymeric surfactants, whose shell around the droplets protect them not only for their amphiphilic nature, but also by steric hindrance [28].

The use of chitosan as absorption enhancer in pharmaceutical delivery systems is common, mainly due to its mucoadhesion and drug permeation enhancer properties. An important contribution to mucoadhesion is represented by the ionic interaction with the anionic sialic acid moieties of mucins, the characteristic glycoproteins of the mucus. This interaction depends on the chitosan positive charge due to the presence along the polymer chain of free amino groups in the ionized status. It is influenced therefore by deacetylation degree and by environmental pH. The role of chitosan as an absorption enhancer has been also deeply studied, both in nasal [14,29] and in intestinal epithelia [30,31]. About the mechanisms involved in this effect, clear evidence has been provided to support the chitosan ability to open the tight junctions [32], thus decreasing paracellular resistance. However, also a mechanism involving the activation by chitosan of a chloride-bicarbonate exchanger has been pointed out to explain its reduction effect on transcellular resistance [33]. Both these mechanisms have been anyway attributed to the cationic form of the polymer.

Permeation enhancement properties are also attributed to oleic acid. This effect, observed after dermal and transdermal applications, has been attributed to modification of lipid layers and cell membranes. Some papers, however, pointed out the ability of oleic acid to open tight junctions by interfering with calcium ions [16]. The ability of oleic acid to open the tight junctions was also observed by Kobayashi et al. in A549 cell lines [34]. 

Based on the above mentioned chitosan and oleic acid properties, the G-CS-OA-2.0% sample appeared therefore optimal in order to improve geraniol absorption following its oral administration. Therefore, this sample has been selected to orally administer geraniol in rats and to perform pharmacokinetic studies. Moreover, taking into account the potential therapeutic prospective of geraniol in CNS pathologies, the nasal administration of the G-CS-OA-2.0% sample has been also evaluated as putative administration strategy to improve brain geraniol uptake.

Different dosages to rats were chosen for the geraniol administration: firstly, for the oral route we chose 50 mg/kg, often used for therapeutic studies [5,6,7,10]. A dosage about ten times lower (4 mg/kg, i.e., 1 mg/rat) was also chosen in order to investigate the potential geraniol uptake in the CFS, following the nasal administration. This lower nasal dosage in comparison with the oral administration was in line with the dose reductions that we have previously adopted for nasal administration of rokitamycin or deferoxamine [35,36]. Moreover, taking into account the potential absorption enhancement properties of the G-CS-OA-2.0% sample, we chose also to test the reduced dose to 1 mg/kg for both oral and nasal administrations in order to perform a comparison between these two ways. This last dose was normally used by us for nasal administration of several neuroactive drugs or prodrugs [37,38,39,40]. 

The oral administration of the 50 mg/kg dose allowed to obtain in rat blood an AUC value of 42,713 ± 1553 µg⋅mL^−1^ min. This value is about six-fold higher than that (6911 ± 279 µg⋅mL^−1^ min) we previously obtained by administering, under the same experimental conditions, a coarse emulsion of geraniol in presence of glycerol [12]. Currently, the reason for this surprising result could not be pinpointed, but some speculations could be proposed. Firstly, it can be attributed to both the possible higher stability and/or homogeneity of the C-CS-OA-2.0% sample in comparison of the coarse emulsion formulated in the presence of glycerol, possibly allowing, especially at high dosages, a better dose precision than the coarse emulsion formulation. In fact, we observed that the present G-CS-OA-2.0% formulation stored three months at 4 °C did not significantly change during time, while the coarse emulsion used in the previous study [12] displayed a quite low stability (unpublished data). This hypothesis is supported by the evidence that, due to is chemical characteristics, geraniol requires specific formulations to be orally administered [7]. Moreover, the CS-OA 2.0% sample probably induces an increased geraniol contact area with the gastrointestinal mucosa than the coarse emulsion [41,42]. Here, the mucoadhesive and absorption enhancement properties of chitosan-oleate can finally amplify the geraniol absorption in the bloodstream in comparison with the coarse and unstable emulsion obtained in the presence of glycerol. An analogous result, as obtained in the present study, with nanoemulsions [41] was explained considering the high contact surface of the disperse phase with the absorption mucosa but also supposing an inhibition of efflux transporters like the P-gp pump. In the present case, although dimensions of the dispersed phase were found slightly below the micrometer, the surface area is in any case quite large with respect to coarse emulsions. Moreover, it is reported that fatty acids may increase the oral absorption of several substances by interfering with some apical efflux transporters [43], so that also this mechanism could be considered as a potential explanation of the results observed. However, other mechanism(s) might be involved in the observed high geraniol absorption after its oral administration in G-CS-OA 2.0% formulation and further studies are necessary to clarify this aspect. It is worth noting that this effect does not appear limited to the intestinal mucosa, but seems also to contribute to increase the geraniol uptake in the CNS from the bloodstream. Indeed, the oral administration to rats of 50 mg/kg geraniol in G-CS-OA-2.0% formulation allowed in the CSF an AUC value of 7293 ± 408 µg⋅mL^−1^ min, showing a CSF/blood ratio of about 0.2. Interestingly, the AUC value obtained in the rat CSF, by administering geraniol as coarse emulsion under the same experimental conditions, was 72.85 ± 3.45 µg⋅mL^−1^ min [12]. In that case the CSF/blood ratio was 0.01, about 20 times lower that that obtained in the present study. The ratio value of about 0.2 was obtained also with the oral administration of 1 mg/kg of geraniol by using the G-CS-OA-2.0% formulation, confirming the aptitude of this type of emulsion as promoter of geraniol uptake in the CNS from the bloodstream. This is a quite peculiar result; whose mechanisms have to be better clarified. 

The nasal administration of G-CS-OA-2.0% sample reversed the CSF/blood AUC ratio of geraniol, being 6.5 and 13.3 for the 4 mg/kg and 1 mg/kg doses, respectively. In particular, this last dose allowed to obtain in the CSF an AUC value of 5571 ± 290 µg⋅mL^−1^ min, about 15 times higher than the AUC value (399 ± 25 µg⋅mL^−1^ min) obtained by oral administration of the same dose. These results evidence the opportunity in choosing the nasal way in order to improve the geraniol uptake in CNS. 

Literature is quite rich of examples of nose to brain delivery through the formulation of poorly soluble drugs in microemulsion and nanoemulsion formulations [44]. In many of them chitosan was associated with the formulation, and its mucoadhesive behavior is indicated as one of the factors responsible for the success in terms of bioavailability improvement [45,46]. The ability of chitosan to open the tight junctions seems another possible reason for the improvement of absorption that should especially occur in this case by paracellular route. Absorption enhancement by membrane fluidification could moreover be attributed to the surfactant components of most of the nanoemulsions described in the literature [47]. In the present case, the obtained results can be attributed to chitosan actions, as mucoadhesive polymer and as absorption enhancer. Oleic acid can play a further role as absorption enhancer, although in this case it has still to be understood if either the opening the tight junctions or the membrane effect due to lipidic disturbance is likely to have higher relevance. Although the dimensional characterization of the sample tested suggests a quite large distribution ranging from a few hundred nanometers to few microns, it seems less likely that the finest fractions are responsible for absorption improvement through endocytosis mechanisms. The fine dispersion however is likely to play an important role in determining a particularly good interaction of the oil droplets with the mucosal sites, both nasal and oral. 

In terms of safety concerns, toxicity studies about the systems require a further, more exhaustive evaluation, that could be the subject of future work. However, CS-OA demonstrated good biocompatibility towards human fibroblasts and keratinocytes as reported in some previous studies [17,18,21], and towards Hep-2, Caco-2, and WKD cell lines, as reported in Bonferoni et al. [20]. It must be noted that CS-OA is not obtained by any covalent reaction, but it is based on an electrostatic interaction between chitosan and oleic acid, both largely used in different pharmaceutical applications. Although it has been observed that this interaction is to some extent sensitive to medium ions [17], the relevance of this effect on absorption is still under study. 

## 5. Conclusions

Despite the interesting potential therapeutic properties of geraniol, its aptitude to easily separate from aqueous formulations can often affect the precision of its administration and induce mucosal irritation. Here, we demonstrate that an emulsion in water of geraniol can be easily obtained in the presence of chitosan oleate as surfactant. In particular, a homogenous distribution of this highly lipophilic compound as droplets with diameters lower than 1 µm was obtained, showing high stability during time. The oral administration of this formulation induced not only a great increase of geraniol bioavailability, but also an enhancement of its aptitude to target the CNS from the bloodstream in comparison to formulations constituted by coarse geraniol emulsion. Moreover, appreciable geraniol amounts were detected in the CNS following the nasal administration of relatively low doses of the chitosan oleate emulsion of geraniol. 

The results presented, although preliminary, suggest that the encapsulation in chitosan oleate of drugs with low solubility leads to a remarkable improvement of bioavailability. Further studies are needed to understand the mechanisms of the observed improvement after both nasal and oral delivery, also using preparations with reduced dimensions of the dispersed phase, to understand the role of the nanometric portion in the bioavailability enhancement. Efforts should be made to clarify the effect on mucociliary activity and on mucosal integrity.

## Figures and Tables

**Table 1:** AUC values obtained in the blood and CSF of rats after oral or nasal administration of different doses of geraniol formulated in the sample G-CS-OA-2.0%. The table reports the CSF/blood ratio of AUC values obtained for each type of administration. The AUC values are reported as the mean ± S.D. of at least four independent experiments.

**Oral 50 mg/kg**			**Nasal 4 mg/kg**		
	**µg∙mL^−1^∙min**	**CSF/Blood Ratio**		**µg∙mL^−1^∙min**	**CSF/Blood Ratio**
Blood	42,713 ± 1553	0.17	Blood	1668 ±101	6.5
CSF	7293 ± 408 ^1^		CSF	10,778 ± 477 ^1^	
**Oral 1 mg/kg**			**Nasal 1 mg/kg**		
	**µg∙mL^−1^∙min**	**CSF/Blood Ratio**		**µg∙mL^−1^∙min**	**CSF/Blood Ratio**
Blood	2158 ± 82	0.18	Blood	419 ± 23	13.3
CSF	399 ± 25 ^2^		CSF	5571 ± 290 ^1^	

^1^*p* < 0.001 versus blood; ^2^ *p* < 0.05 versus blood.
